# Human placental trophoblast cells contribute to maternal–fetal tolerance through expressing IL-35 and mediating iT_R_35 conversion

**DOI:** 10.1038/s41467-019-12484-z

**Published:** 2019-10-10

**Authors:** Jia Liu, Shengnan Hao, Xi Chen, Hui Zhao, Lutao Du, Hanxiao Ren, Chuanxin Wang, Haiting Mao

**Affiliations:** Department of Clinical Laboratory, The Second Hospital of Shandong University, 250033 Jinan, Shandong Province People’s Republic of China

**Keywords:** Peripheral tolerance, Interleukins, Regulatory T cells, Signal transduction

## Abstract

During pregnancy, trophoblast cells sustain the maternal–fetal tolerance via expressing and secreting various chemokines and cytokines. Our previous study revealed the expression of interleukin-35 (IL-35) in human first-trimester trophoblasts. Here we show that IL-35 is expressed in both human first-trimester primary trophoblast cells and a trophoblast cell line. Trophoblast cells inhibit the proliferation of human naive conventional T cells (T_conv_ cells) and convert suppressed T_conv_ cells into iT_R_35 in an IL-35-dependent manner. Mechanistically, trophoblast cell derived IL-35 mediates its function through phosphorylation of STAT1 and STAT3. In vivo studies confirm that mice with immunologically spontaneous abortion have lower levels of IL-35 and iT_R_35 cells at the maternal–fetal interface, and neutralizing anti-IL-35 mAb enhances abortion rates. Meanwhile, exogenous IL-35 induces iT_R_35 and prevents immunological abortion. Our findings thus suggest that trophoblast cells have a critical function in preserving maternal–fetal tolerance via secreting IL-35 during pregnancy.

## Abstract (web summary)

Trophoblasts play a critical role in maintaining immunological tolerance at maternal–fetal interface. Here the authors show that IL-35 is made by trophoblasts, converts T cells to immune tolerant iTR35 cells, and is critical to prevent spontaneous abortion.

## Introduction

Immunologically, the fetus is considered as an allograft that resides in an immune-competent mother and the maternal immune system must tolerate this allogenic fetus to maintain a successful pregnancy^1^. The induction of maternal–fetal tolerance is achieved by synergic action of cells and cytokines in maternal–fetal interface^2,3^. However, the mechanism underlying this unique immunological behavior remains poorly understood. As a key component in the human placenta, trophoblast cells express and secrete various chemokines and cytokines in addition to their proliferative and invasive properties, thereby acting as effector cells in sustaining maternal–fetal tolerance^4^. For instance, trophoblast cells selectively recruit peripheral immune cells, such as natural killer and T cells, to the decidua via secreting a cascade of strictly controlled chemokines (CXCL12, CXCL16, and CCL3) and then modulate their function^5,6^. Trophoblast cells also shift the Th1/Th2 ratio toward Th2 and inhibit Th17 immunity at fetomaternal interface via secreting cytokines such as IL-10 and thymic stromal lymphopoietins (TSLPs)^4,7^. Our previous study showed that immunosuppressive cytokine IL-35 was constitutively expressed in human first-trimester trophoblasts^8^.

As the newest member of IL-12 cytokines family, IL-35 potentially suppresses proliferation and activation of Th1 and Th17 cells in a context-dependent manner while facilitating suppressor function of regulatory T cells (Tregs) in a number of auto-immune diseases. IL-35 also plays key role in modulating the ratio of M1/M2 macrophages and inducing the tolerogenic phenotype on dendritic cells^9^. Another important function of IL-35 is that it converts naive conventional T cells (T_conv_ cells) into IL-35-producing induced regulatory T cells, referred as iT_R_35^10,11^. Given the immune suppressive role of IL-35 and its consecutive expression in trophoblast cells, we speculate that IL-35 secreted by trophoblast cells may participate in maternal–fetal tolerance through modulating T_conv_ cells proliferation and differentiation.

In this study, we show that IL-35 from trophoblast cells suppress the proliferation of T_conv_ cells and further convert them into iT_R_35. We also characterize the phenotype of this regulatory population and address the key transcription factors by which trophoblast cells derived-IL-35 mediates the conversion. In addition, physiological contribution of IL-35 on maternal–fetal tolerance is further assessed in vivo.

## Results

### IL-35 expression in human serum and trophoblast cells

**Fig. 1:**
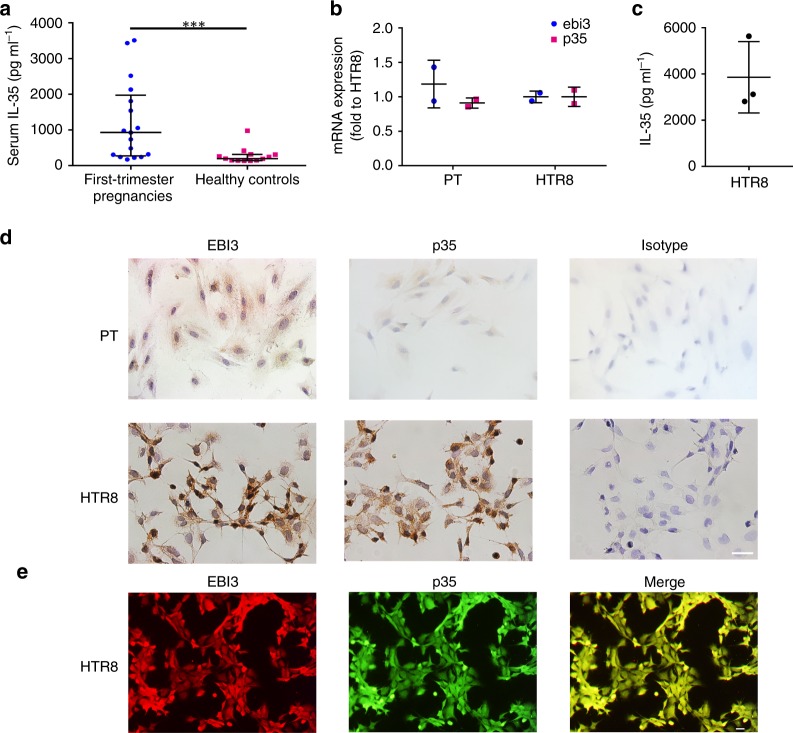
IL-35 is present in the human serum and trophoblast cells. **a** The serum from early pregnant women (left, *n* = 17) and age matched non-pregnant women (right, *n* = 13) were collected and IL-35 level was measured by ELISA. The detection range of IL-35 was 62.5–4000 pg ml^−1^. **b** The mRNA level of the two subunits of IL-35 (*ebi3* and *p35*) in primary trophoblasts (PT) and HTR8 cells were detected by quantitative real-time RT-PCR analysis. The results were normalized to endogenous control (*GAPDH*) (*n* = 2). **c** ELISA of IL-35 content in the supernatant of HTR8 cells (*n* = 3). **d** Immunocytochemical staining of EBI3 and p35 in PT and HTR8 cells. Scale bar: 50 μm. **e** Immunofluorescence staining of EBI3 (red) and p35 (green) in HTR8 cells. Scale bar: 20 μm. Data in 1a were represented as median with interquartile range. Significance was determined by Mann–Whitney *U*-test. Data in 1b and 1c were represented as mean ± SD. Significance was determined by Student’s *t* test analysis. ***P < 0.001

We first measured IL-35 level in the peripheral blood from early pregnant women and age matched non-pregnant women by ELISA. Significant upregulation of serum IL-35 level was observed in the first trimester of pregnancies compared with non-pregnant healthy controls (Fig. 1a). Subsequently, we investigated the expression of IL-35 in primary trophoblast cells (PT) and trophoblast cell line-HTR8. The results of real-time RT-PCR displayed the expression of *EBI3* and *p35* on the mRNA level in PT and HTR8 cells (Fig. 1b). Furthermore, quantitive analysis by ELISA determined the content of IL-35 as 3857 pg ml^−1^ in the culture supernatant of HTR8 cells (Fig. 1c). By performing immunocytochemical staining, we demonstrated that both PT and HTR8 cells constitutively expressed the two subunits of IL-35, EBI3, and p35 (Fig. 1d). Further evaluation using immunofluorescence showed that both of the two subunits co-located in the cytoplasm of trophoblast cells (Fig. 1e). Therefore, first trimester trophoblast cells are able to express and secrete immunosuppressive cytokine IL-35.

### Trophoblast cells-derived IL-35 inhibits T_conv_ proliferation

**Fig. 2:**
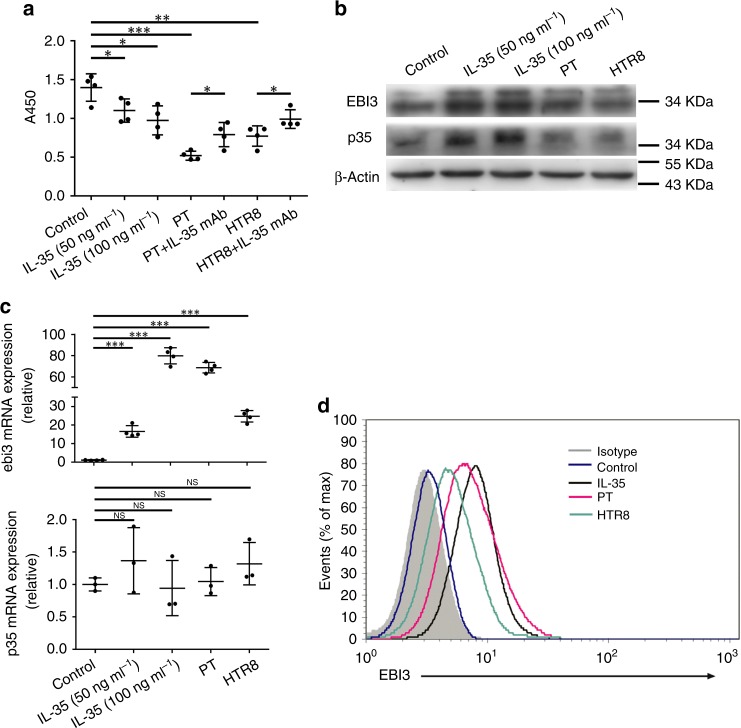
Trophoblast cells inhibit the proliferation of T_conv_ and mediate its conversion via secreting IL-35. T_conv_ cells were cultured in medium (control) alone, or with human r-sc-IL-35 (IL-35) (50 ng ml^−1^ or 100 ng ml^−1^), or with trophoblast cells supernatant (with or without neutralizing anti-IL-35 mAb) in presence of anti-CD3/anti-CD28 beads and IL-2 for 5 days. **a** Then, the proliferation of T_conv_ cells was evaluated by CCK8 assay (*n* = 4). **b** The protein levels of EBI3 and p35 in treated T_conv_ cells were determined by western blot (*n* = 3). **c** The mRNA levels of *ebi3* (*n* = 4) and *p35* (*n* = 3) in T_conv_ cells were detected by quantitative real-time RT-PCR. The results were normalized to endogenous control (*GAPDH*). **d** Flow cytometric analysis of EBI3 in treated T_conv_ cells. Cells were harvested and stained with anti-EBI3 or isotype-matched control antibody (Isotype) after activated with Cell Activation Cocktail (*n* = 3). Data were represented as mean ± SD. Significance was determined by Student’s *t* test analysis. *P < 0.05, **P < 0.01, and ***P < 0.001

To explore the function of trophoblast cells-derived IL-35 in maintenance of pregnancy, purified T_conv_ cells were treated with human r-sc-IL-35, trophoblast cells supernatant or neutralizing anti-IL-35 mAb in presence of anti-CD3/anti-CD28 beads and IL-2. Results of CCK8 assay revealed that supernatant from PT or HTR8 cells dramatically inhibited the proliferation of T_conv_ cells similar to that induced by r-sc-IL-35. However, neutralizing anti-IL-35 mAb partly blocked the suppressive capacity of trophoblast cells supernatant (Fig. 2a). These results indicate that, although without direct cell contact, trophoblast cells suppress the proliferation of T_conv_ cells via secreting IL-35.

### Trophoblast cells-derived IL-35 converts T_conv_ into iT_R_35

Next, we investigated whether trophoblast cells-derived IL-35 could induce the conversion of T_conv_ cells into iT_R_35. As shown in Fig. 2b, c, compared with control group, the supernatant from PT or HTR8 cells substantially upregulated the IL-35 expression of T_conv_ cells on both mRNA and protein levels. We also observed that mRNA and protein levels of *p35* subunit were inconsistent in different groups and this might be explained by post-transcriptional and translational regulation, such as alternative splicing and mRNA decay^12^. Single-cell analysis by intracellular cytokine staining further revealed that treatment with human r-sc-IL-35 or trophoblast cells supernatant, all induced the significantly increased expression of IL-35 in T_conv_ cells (Fig. 2d). Collectively, these data suggest that trophoblast cells-derived IL-35 converts T_conv_ cells into iT_R_35.

### Microarray analysis of T_conv_ induced by trophoblast cells

**Fig. 3:**
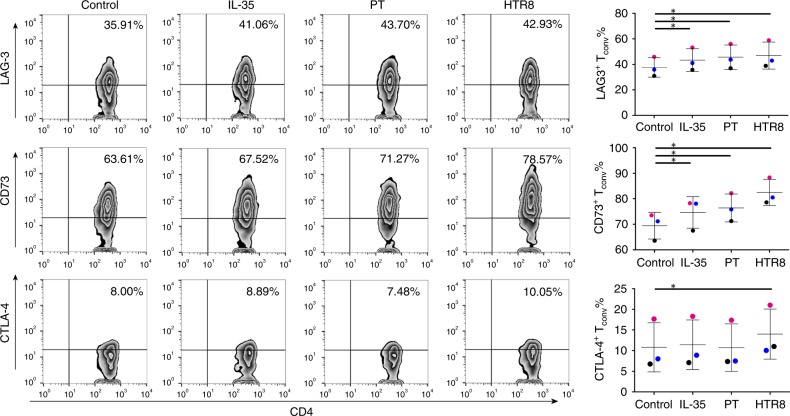
Inhibitory phenotypic analysis of trophoblast cells-induced iT_R_35 cells. T_conv_ cells were cultured in medium alone, or with IL-35 or supernatant from trophoblast cells for 5 days. Then cells were harvested for flow cytometry analysis to detect the surface molecules including CTLA-4, CD73, and LAG-3. Density plots showing percentages of CTLA-4^+^, CD73^+^, and LAG-3^+^ cells among T_conv_ cells (left) and the corresponding statistical analysis (right) (*n* = 3). Data were represented as mean ± SD. Significance was determined by paired Student’s *t* test analysis. *P < 0.05

Given the results aforementioned that trophoblast cells-derived IL-35 inhibited the proliferation of T_conv_ cells and converted them into suppressive iT_R_35 cells, we next sought to define their phenotypes. After treatment with r-sc-IL-35 or trophoblast cells supernatant for 5 days, T_conv_ cells were collected and stained with fluorescence-conjugated monoclonal antibodies for flow cytometry analysis. The results showed that inhibitory molecules including LAG-3 and CD73 were visibly upregulated in T_conv_ cells treated with r-sc-IL-35 and the supernatant from PT or HTR8 cells. However, a slight increase in CTLA-4 expression was observed only in T_conv_ cells stimulated with the supernatant of HTR8 cells (Fig. 3).

**Fig. 4:**
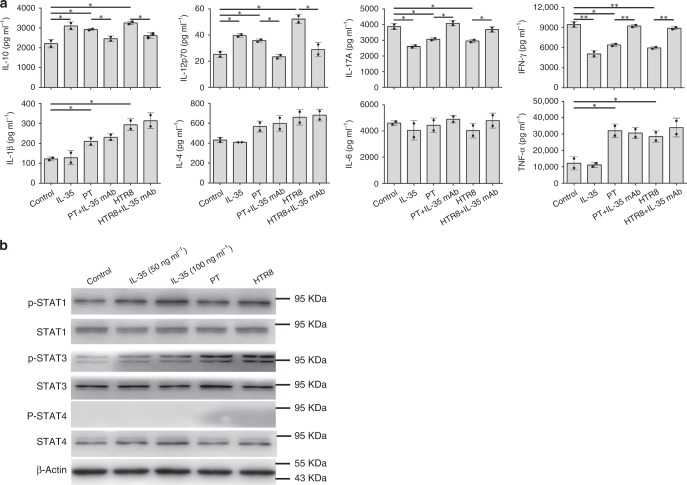
Cytokines secretion profile evaluation of T_conv_ cells induced by trophoblast cells-derived IL-35 and the related transcription factors which mediate the conversion. **a** T_conv_ cells were cultured in medium (control) alone, or with human r-sc-IL-35 (IL-35) (50 ng ml^−1^), or with trophoblast cells supernatant (with or without neutralizing anti-IL-35 mAb) for 5 days. Then the supernatants of activated T_conv_ cells were collected for cytokines secretion profile evaluation using Bio-Plex Protein Array system. In T_conv_ cells co-cultured with supernatant from PT or HTR8 cells, final results of cytokines concentration were normalized to the initial supernatant from PT or HTR8 cells, respectively, which were used as inner control (*n* = 2). **b** T_conv_ cells were cultured in medium (control), or with human r-sc-IL-35 (IL-35) (50 ng ml^−1^ or 100 ng ml^−1^), or with trophoblast cells (PT and HTR8) supernatant for 1 h. Then, T_conv_ cells were collected and the protein levels of phosphorylated (p) and total STAT1, STAT3, and STAT4 were determined by western blot (*n* = 3). Data were represented as mean ± SD. Significance was determined by Student’s *t*-test. *P < 0.05 and **P < 0.01

On the other hand, cytokine secretion profile of converted T_conv_ cells was also evaluated by Bio-Plex Protein Array system. We observed a significant increase of IL-10 and IL-12p70 level, and obviously decrease of IL-17A and INF-γ in T_conv_ cells treated with r-sc-IL-35 or supernatant of trophoblast cells compared with control group. Moreover, the addition of a functional anti-IL-35 mAb markedly blocked these effects of trophoblast cells supernatant. Notably, levels of cytokines, such as IL-1β and TNF-α, were also significantly elevated in trophoblast cells supernatant groups in an IL-35-independent manner (Fig. 4a).

### Key transcription factors in the induction of iT_R_35

As IL-35 belongs to IL-12 family of cytokines which signals through STAT family of transcription factors^13^, we further investigated which STAT proteins were associated with iT_R_35 induced by trophoblast cells-derived IL-35. Western blot analysis showed that treatment of T_conv_ cells in the presence of r-sc-IL-35 or trophoblast cells supernatant for 1 h resulted in robust phosphorylation of STAT1 and STAT3, whereas p-STAT4 was not detected (Fig. 4b). These findings demonstrate that trophoblast cells-derived IL-35 exerts its biological function on T_conv_ cells through the activation of transcription factors STAT1 and STAT3.

### Contribution of IL-35 and iT_R_35 cells to pregnancy in vivo

**Fig. 5:**
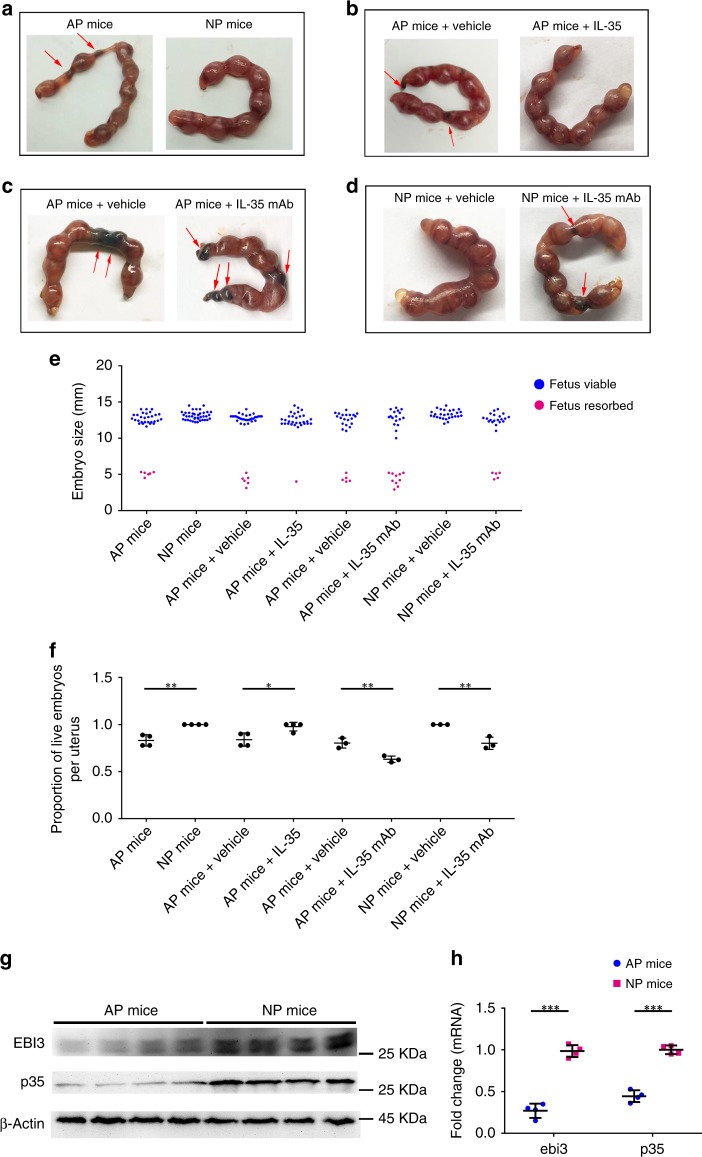
Phenotype of fetuses from NP mice and AP mice and the expression of IL-35 in the placenta. Pregnant CBA/J female mice were euthanized on Day 13.5 of pregnancy. **a**–**d** Representative pictures showing the embryos and live fetuses per uterus from different treated AP and NP mice. Red arrows: aborted embryos. **e** Embryos size were measured. From left to right: *n* = 35 embryos from four separate mothers (AP mice), *n* = 39 embryos from four separate mothers (NP mice), *n* = 38 embryos from four separate mothers (AP mice + vehicle), *n* = 31 embryos from four separate mothers (AP mice + IL-35), *n* = 25 embryos from three separate mothers (AP mice + vehicle), *n* = 27 embryos from three separate mothers (AP mice + IL-35 mAb), *n* = 28 embryos from three separate mothers (NP mice + vehicle) and *n* = 25 embryos from three separate mothers (NP mice + IL-35 mAb). **f** Proportion of live embryos per uterus was calculated with following formula: Proportion of live embryos per uterus = (Number of live embryos per uterus)/(Number of total embryos per uterus). **g**–**h** Pregnant CBA/J female mice were euthanized on Day 13.5 of pregnancy and the placenta was isolated and teased apart. Then the protein and mRNA were extracted from the tissue. The protein levels of EBI3 and p35 in placenta were determined by western blot (*n* = 4) (**g**). Quantitative real-time RT-PCR analysis was applied to compare the expression of *ebi3* and *p35* in the placenta of NP and AP females (*n* = 4) (**h**). Data were represented as mean ± SD. Significance was determined by Student’s *t*-test. *P < 0.05, **P < 0.01, and ***P < 0.001

**Fig. 6:**
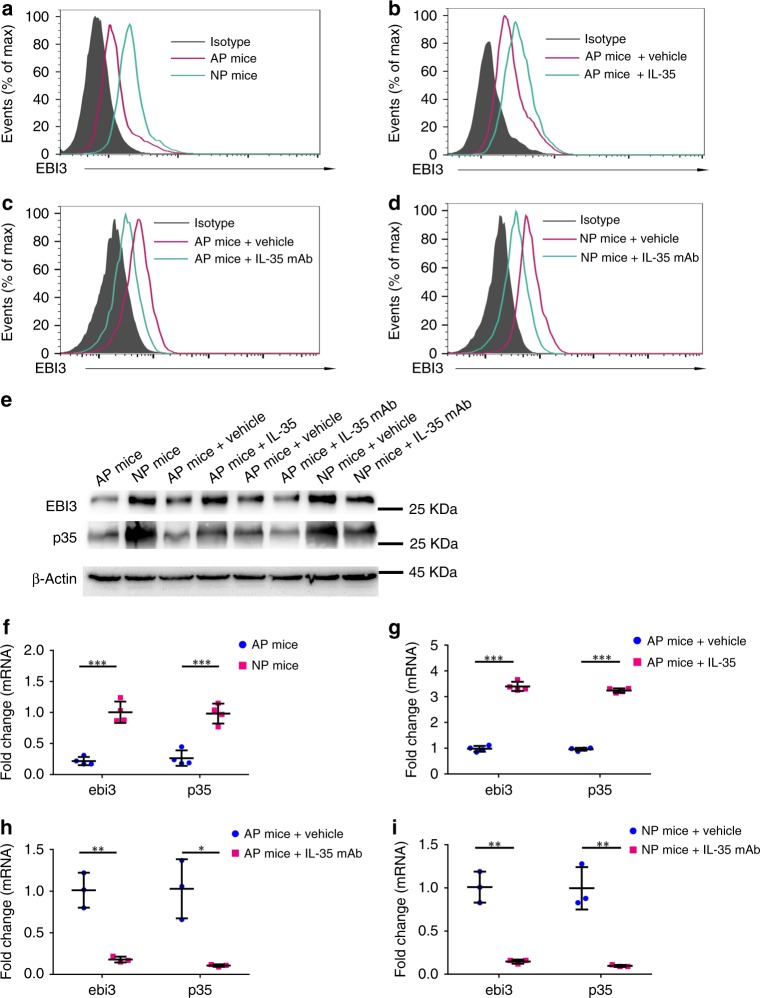
IL-35 expression pattern in T_conv_ cells from different treated NP or AP mice. Pregnant CBA/J female mice were euthanized on Day 13.5 of pregnancy, the decidua was isolated and teased apart. **a**–**d** T_conv_ cells were isolated from the decidua and stained with anti-EBI3 or isotype-matched control antibody (Isotype) for flow cytometry analysis. **e** The protein levels of EBI3 and p35 in T_conv_ cells were determined by western blot. **f**–**i** The mRNA level of *ebi3* and *p35* in decidual T_conv_ cells were analyzed using quantitative real-time RT-PCR analysis. The results were normalized to endogenous control (*GAPDH*). **f** *n* = 4 for AP mice group, *n* = 4 for NP mice group. **g** *n* = 4 for AP mice + vehicle group, *n* = 4 for AP mice + IL-35 group. **h** *n* = 3 for AP mice + vehicle group, *n* = 3 for AP mice + IL-35 mAb group. **i** *n* = 3 for NP mice + vehicle group, *n* = 3 for NP mice + IL-35 mAb group. Data were represented as mean ± SD. Significance was determined by Student’s *t*-test. *P < 0.05, **P < 0.01, and ***P < 0.001

To evaluate the role of IL-35 secreted by trophoblast cells in maintaining maternal–fetal immune-tolerance, a well-described murine model of immunological spontaneous abortion was established. In this model, an abnormal maternal immune response led to the rejection of the fetuses and spontaneous abortion in CBA/J × DBA/2 J group (AP), but not in CBA/J × BALB/C group (NP) (Fig. 5a, e, f). First, the expression of IL-35 in the placenta was compared between AP and NP females. We observed an obviously downregulation of IL-35 mRNA and protein levels in AP females versus NP females (Fig. 5g, h). Next, T_conv_ cells were isolated from the decidua for phenotype analysis and IL-35 expression evaluation. According to the results of single-cell analysis, NP females experienced a higher expression of IL-35 in decidual T_conv_ cells than AP females (Fig. 6a). Similarly, higher levels of IL-35 mRNA and protein were also observed in the decidual T_conv_ cells of NP females than that in AP females (Fig. 6e, f).

Having demonstrated that IL-35 and iT_R_35 cells were augmented in NP but not in AP females, we next assessed the therapeutic effect of IL-35 on immunological abortion in AP females. Firstly, AP mice were randomly divided into two groups: the control group received phosphate buffer saline (PBS) injection (vehicle); the experiment group received mouse r-sc-IL-35 injection. As supported by Fig. 5b, e, f, treatment of mouse r-sc-IL-35 markedly decreased the fetal resorption rate of AP group. And then we confirmed that decidual T_conv_ cells from r-sc-IL-35-treated AP females showed elevated IL-35 mRNA and protein expression than control group (Fig. 6e, g), which was in consistence with the results of flow cytometry (Fig. 6b). In addition, neutralizing IL-35 antibody was also administered intraperitoneally to both NP and AP females in order to further evaluate the effect of IL-35. The results displayed that treatment of anti-IL-35 mAb enhanced abortion rates in both matings (Fig. 5c–f). Flow cytometry analysis demonstrated that anti-IL-35 mAb decreased the IL-35 expression of T_conv_ cells in both NP and AP females (Fig. 6c, d). Similar results were also detected at the mRNA and protein levels (Fig. 6e, h, i). Taken together, these findings indicate that trophoblast cells-derived IL-35 promotes the expansion of mice decidual iT_R_35 cells in pregnancy. Immunologically spontaneous abortion mice have lower levels of IL-35 and iT_R_35 cells and exogenous IL-35 treatment helps to prevent abortion.

## Discussion

During pregnancy, maintenance of maternal–fetal tolerance requires a complicated network of communications among trophoblast cells, decidual stromal cells and immune cells^14^. Particularly, an important role is assigned to the bidirectional communications between trophoblast cells and immune cells^15^. In this cross talk, trophoblast cells secret a series of cytokines to participate in the recruitment of immune cells such as T cells and further modulate their proliferation and polarization^16,17^. Notably, as we have previously depicted, human placental villi constitutively expressed inhibitory cytokine IL-35 and IL-35 secretion was also detectable in PT cells under physiological conditions^8^. In this study, we first demonstrated IL-35 expression in human first-trimester primary trophoblast cells and trophoblast cell line, and the serum IL-35 level of early pregnant women obviously increased. All these suggested that IL-35 might play an important role in maintenance of maternal–fetal tolerance.

IL-35 is initially described as an immunosuppressive cytokine specifically produced by natural Treg and is required for maximal Treg function in vitro and in vivo^18^. Moreover, IL-35 from Treg mediates the proliferation suppression of T_conv_ cells and the induction of iT_R_35^19^. In the present study, we also evaluated the function of trophoblast cells-derived IL-35 on T_conv_ cells. Similar effects were observed as IL-35 secreted by trophoblast cells (both PT and HTR8) obviously inhibited the proliferation of T_conv_ cells. Although previous studies have already demonstrated that supernatants from human trophoblastic choriocarcinoma cell lines inhibit T cell proliferation^20,21^, suppressor factors in the trophoblast cells supernatant are not identified in these studies and the underlying mechanisms still remains largely unknown. Our results here proved for the first time that IL-35 secreted by trophoblast cells suppressed T cell proliferation and induced the conversion of T_conv_ cells into iT_R_35.

Subsequently, we analyzed the phenotype and cytokine secretion profile of IL-35-treated T_conv_ cells. Surface molecules including LAG3, CD73, and CTLA-4 which have been described as the mediators of nTreg cell suppression were included into analysis^22–24^. Our data showed that trophoblast cells-derived IL-35 induced the upregulation of surface inhibitory receptors LAG3 and CD73 expression on T_conv_ cells as estimated and these molecules were important to maximize the suppressive activity of Tregs. In paralleled with the results of phenotypic analysis, a significant increase in the production of inhibitory cytokine IL-10 was observed in T_conv_ cells induced by IL-35 from trophoblast cells. And this result was consistent with previous report that IL-35 induced the expansion of Treg cell population along with an elevated production of IL-10 ^19^. According to the previous studies, IL-35 directly suppressed Th1 and Th17 cells and resulted in a reduced production of INF-γ and IL-17^25,26^. Here our observation also confirmed that suppression of T_conv_ cells proliferation was accompanied by a dramatic decrease in the secretion of IFN-γ and IL-17A. In addition, other factors besides IL-35 in the trophoblast cells supernatant might also participate in the conversion of T_conv_ cells as the alteration in cytokines, such as IL-1β and TNF-α, had not been attenuated by IL-35 blockage.

Infectious tolerance is thought to play a significant role in propagating Treg-mediated immune tolerance^27^. It has been suggested that TGF-β participates in infectious tolerance as nTreg cells confer a suppressive phenotype on T_conv_ cells via TGF-β dependent pathway^28^. The key role of IL-10 in mediating infectious tolerance with Treg cells was also observed in autoimmune disease^29^. Besides, nTreg cells secreted IL-35 and IL-10 and converted T_conv_ cells into iT_R_35 cells by an IL-35-dependent and IL-10-dependent manner^19,30^. Indeed, our data showed that trophoblast cells-induced iT_R_35 also secreted homogenous IL-35 and IL-10, which suggested the possibility that IL-35 and iT_R_35 cells at maternal–fetal interface might represent additional, importantly mediators of infectious tolerance.

Based on the results aforementioned, we next explored the underlying mechanism and signaling pathways by which trophoblast cells-derived IL-35 exerted its effect. Chain sharing is common in cytokines of IL-12 family which extends to their receptors and transcription factors^31^. Cytokines in IL-12 family generally signals through the phosphorylation and activation of signal transducers and activators of transcription (STAT) family^32^. However, there is diversity in the STAT factors activated by IL-35 in different cell types and different species. Studies in mice verified that IL-35 mediated regulatory function in T_conv_ cells via the phosphorylation of STAT1, STAT3, and STAT4^33,34^ while IL-35 signaling induced STAT1 and STAT3 phosphorylation in murine B cells, which was different from T cells^34^. Here, we confirmed that trophoblast cells-derived IL-35 signaled through the activation of STAT1 and STAT3 in human T_conv_ cells, but no phosphorylation of STAT4 above background and this was in consistence with a previous study which described the role of IL-35 in human colorectal cancers^35^. In this regard, several possibilities were put forward. Firstly, IL-35 might utilize different receptors and signaling components in various species and cell types^34^. Besides, even though the same receptors were adopted by IL-35, the diverse higher-order multimers formed by receptor chains (gp130 and IL-12Rβ2) might still lead to the activation of different transcription factors^33^. Collectively, these data suggested an additional layer of complexity underlying the regulatory role of IL-35, which remained to be further elucidated.

In view of the results in vitro, we further assessed the role of IL-35 and iT_R_35 in maternal–fetal tolerance maintenance by spontaneous abortion mice model. Both trophoblast cells-derived IL-35 and decidual iT_R_35 were significantly decreased in AP females compared with NP females. Exogenous IL-35 administration to AP females attenuated the fetal loss via facilitating decidual iT_R_35 conversion while neutralizing anti-IL-35 mAb suppressed iT_R_35 cells conversion and enhanced abortion rates. This further confirmed that IL-35 was an important factor during normal pregnancy. It has been reported that redundant Th17 cells and diminished Treg, as well as the imbalance of Th1/Th2 cytokines are the main reasons for spontaneous abortion^36–40^. Here, we provided the evidence that reduced production of IL-35 in trophoblast cells and a consequent decrease in the number of decidual iT_R_35 were also the important causes that led to spontaneous abortion. These observations provided important clues for the pathogenesis of abnormal pregnancies and could be useful in further clinical research.

Taken together, our study suggests that trophoblast cells contribute to maternal–fetal tolerance via IL-35 secretion, which could suppress the proliferation and induce the conversion of decidual T_conv_ cells into iT_R_35 at maternal–fetal interface. Although additional experiments are still needed to fully understand the underlying mechanisms, current findings support that trophoblast cells-derived IL-35 plays a critical role in maintaining a successful pregnancy and IL-35 may represent a valid immunotherapeutic strategy for the treatment of recurrent spontaneous abortions.

## Methods

### Samples collection

Human villous tissues and blood samples were collected from selective terminations of first-trimester pregnancies (5–8 weeks) for non-medical reasons. The blood samples of controls were prepared from age-matched healthy female volunteers. Before sample collection, approval was obtained from the Human Investigation Committee of The Second Hospital of Shandong University, and written informed consent was obtained from each subject. All relevant ethical regulations for work with human participants were followed.

### Cytokines and monoclonal antibodies

Human recombinant single chain IL-35 (r-sc-IL-35) and human recombinant IL-2 (rIL-2) were purchased from Peprotech. Neutralizing anti-human IL-35 mAb was purchased from R&D Systems. Mouse recombinant single chain IL-35 (r-sc-IL-35) was purchased from Chimerigen. Neutralizing anti-mouse IL-35 mAb was purchased from MERCK. In immunocytochemical staining, primary antibodies for human EBI3 and p35 were obtained from Novus Biologicals and R&D Systems. In immunofluorescent staining, primary antibodies for human EBI3 and p35 were obtained from Santa Cruz and R&D Systems. In western blot assay, primary antibodies for human p-STAT1, STAT1, p-STAT3, STAT3, p-STAT4, STAT4, and β-actin were purchased from Cell Signal Technology. Primary antibody for human EBI3 was from Santa Cruz and human p35 was from R&D Systems. Primary antibody for mouse EBI3 and p35 were from Abcam. In flow cytometry, FITC-labeled anti-human CD4 mAb; PE-labeled anti-human LAG-3, CD73, CTLA-4, EBI3 mAb, and related isotype control antibodies were products of eBioscience. PE-labeled anti-mouse EBI3 mAb and related isotype control antibody were the product of R&D Systems.

### Cell line and cell culture

Human trophoblast cell line-HTR8/SVneo that derived from first trimester of pregnancy was a kind gift from Dr. Charles Graham (Queens University, Kingston, ON, Canada). Cells were maintained in RPMI 1640 (Life Technologies/Invitrogen) with 10% fetal bovine serum (FBS) and 1% penicillin/streptomycin (Life Technologies/ Gibco) in a humidified incubator at 37 °C with 5% CO_2_.

### Isolation and culture of human primary trophoblast cells

Fresh trophoblast biopsy specimens were minced into small fragments and then incubated with trypsin (Invitrogen) and DNase І (Sigma Aldrich) for 20 min for three cycles at 37 °C in a constant temperature shaker. Then suspension was aspirated and filtered through 100-μm and then 40-μm pores nylon cell strainers. The collected placental cells were further isolated by density gradient centrifugation using Percoll (Biosharp, Pharmacia) at 1200×*g* for 30 min. The suspension with cells between the density markers of 1.049 and 1.062 g ml^−1^ was collected and then resuspended in RPMI 1640 medium supplemented with FBS for 40 min so that the contaminating macrophages to adhere to the Petri dish. Non adherent trophoblast cells were plated on a Matrigel-coated culture surface in a complete 1640 medium in 5% CO_2_ at 37 °C^8^.

### Isolation and culture of human peripheral T_conv_ cells

Human peripheral blood mononuclear cells (PBMCs) were isolated by density gradient centrifugation using Ficoll-Paque Plus (Sigma Aldrich). Conventional T cells (CD4^+^CD25^−^CD45RA^+^CD45RO^−^) were isolated using human naive CD4^+^ T cell isolation kit II (Miltenyi Biotec). Purity was >97% as confirmed by flow cytometry. Purified T_conv_ cells were cultured in RPMI 1640 medium with rhIL-2 and CD3/CD28 T Cell Activator (Stemcell Technologies).

### ELISA detection of IL-35 level

Enzyme-linked immunosorbent assay (ELISA) kit (CUSABIO) was applied to detect the IL-35 level of serum or HTR-8 cells supernatant according to the manufacturer’s instructions. Each sample was analyzed in triplicate and the mean value was measured. The detection range of IL-35 was 62.5–4000 pg ml^−1^.

### RNA isolation and quantitative real-time RT-PCR

Total RNA was isolated from purified cells using the TRIzol reagent (Invitrogen). For human T_conv_ cells, equal amounts of total RNA from each sample were then reverse-transcribed into cDNA using a RevertTra Ace kit (TOYOBO) and real-time RT-PCR was performed using SYBR Green Realtime PCR Master Mix (TOYOBO). The following sequence specific primers were used: (i) the internal control *GAPDH* gene: forward, 5′-GGTGGTCTCCTCTGACTTCAACAG-3′, reverse, 5′-GTTGTTGTAGCCAAATTCGTTGT-3′; (ii) *ebi3* gene: forward, 5′-GCAGCAGACGCCAACGT-3′, reverse, 5′-CCATGGAGAACAGCTGGACAT-3′; (iii) *p35* gene: forward, 5′-CCTTCACCACTCCCAAAAC-3′, reverse, 5′-TGTCTGGCCTTCTGGAGCAT-3′^41^.

For mice T_conv_ cells, RNA was reverse transcribed using ReverTra Ace Kit (TOYOBO) according to the manufacturer’s instructions. Real-time RT-PCR was performed using SYBR Green Realtime PCR Master Mix (TOYOBO) with the following primers: *ebi3* forward: 5′-CGGTGCCCTACATGCTAAAT-3′; *ebi3* reverse: 5′-GCGGAGTCGGTACTTGAGAG-3′; *p35* forward: 5′-CATCGATGAGCTGATG CAGT-3′, *p35* reverse: 5′-CAGATAGCCCATCACCCTGT-3′. *GAPDH* forward: 5′-AGGTCGGTGTGAACGGATTTG-3′; *GAPDH* reverse: 5′- TGTAGACCATGTA GTTGAGGTCA-3′. *GAPDH* was applied as standard for data normalization.

### Western blot analysis

The protein in cells was extracted using RIPA buffer (Beyotime) and evaluated using the BCA Protein Assay Kit (Beyotime). Equal amounts of protein were separated by SDS-PAGE and transferred to polyvinylidene fluoride (PVDF) membrane (Merck Millipore). Then the membranes were blocked with 5% nonfat dried milk and incubated with primary antibodies overnight at 4 °C. After that, the bands were probed with secondary antibody (ICLLab) and visualized by chemiluminescence (Life Technology). All the uncropped blots were included in the Source Data file.

### CCK8 assay

T_conv_ cells were seeded in triplicate in 96-well plates at 1 × 10^5^ cells per well with human r-sc-IL-35 or the supernatant of primary trophoblasts (PT) and HTR8 cells at a volume 30% of the total culture volume in the presence of neutralizing anti-IL-35 mAb (10 μg ml^−1^) or not for 5 days. Fresh conditional medium was added every 2 days. After treatment, 20 μl of CCK8 solution (Dojindo) was added to each well for the last 2 h. The absorbance value was detected at 450 nm wavelength by a Microplate Reader (Bio-Rad, USA). Results were representative of three individual experiments.

### Immunocytochemical and immunofluorescent staining

IL-35 expression was detected using the avidin-biotin-peroxidase complex method as we previously depicted^8^. Cells with 70% confluence in 6-well plates were fixed in cold acetone/methanol (1:1) and permeabilized with 0.5% Triton X-100. After blocked with 5% bovine serum albumin for 1 h at 37 °C, samples were incubated with anti-EBI3 or anti-p35 antibody at 4 °C overnight. Labeling was detected by adding biotin labeled secondary antibodies, avidin-biotin complex, and stained with DAB. A mouse isotype-matched irrelevant IgG was used as the negative control. For immunofluorescent staining, cells were firstly treated with formaldehyde fixative solution and incubated at room temperature for 20 min. After non-specific staining blocking, cells were probed with anti-EBI3 or anti-p35 antibody. EBI3 was visualized with Alexa 594 (red) and p35 was visualized with Alexa 488 (green). Visualization was conducted using a fluorescence microscope (Olympus, Japan).

### Cytokine measurement

Supernatants from T_conv_ cells treated with stimuli (r-sc-IL-35, PT or HTR8 cells supernantant) were harvested at the indicated time-points and stored at −80 °C until assessment. Cytokine production of IL-1β, IL-4, IL-6, IL-10, IL-12p70, IL-17A, TNF-α, and IFN-γ were simultaneously measured by the Human High Sensitivity Panel (eBioscience) according to the manufacturer’s instructions and each sample was assessed in duplicate.

### Flow cytometry

Cells were harvested and stained for cell-surface markers with monoclonal antibodies against LAG-3, CD73, CTLA-4, and CD4 or their specific isotype controls. For the intracellular staining of IL-35, cells were first pre-incubated with Cell Activation Cocktail (R&D systems), and then fixed and permeabilized with intracellular fixation & permeabilization buffer set (eBioscience) according to the manufacturer’s instructions. Then the cells were incubated with mouse or human EBI3 mAb and analyzed using a BD Biosciences FACS Calibur flow cytometer. Data analysis was performed using FCS express V3 or Flow Jo V10. Gating strategies were presented in Supplementary Fig. 1.

### Animals and animal experiments setup

Eight-week-old CBA/J females, as well as BALB/c and DBA/2 J males were purchased from Institute of Laboratory Animal Sciences, Chinese Academy of Medical Sciences (CAMS). CBA/J × DBA/2 J represents the abortion-prone group (AP), and CBA/J × BALB/c represents the normal pregnancy controls (NP)^42,43^. After the appearance of the vaginal plug, which indicates Day 0.5 of pregnancy, male mice were separated from the females. Five female mice were mated with one male mouse and 3–4 female mice were confirmed to be pregnant in every experiment. Pregnant CBA/J female mice were euthanized on Day 13.5 of pregnancy. For in vivo study of IL-35 treatment, mouse r-sc-IL-35 was administered i.p. (0.75 μg per day, dissolved in 300 μl of PBS) to the AP mice from Day 2 to Day 12 of pregnancy. The control AP mice were injected with 300 μl of PBS only. For in vivo study of neutralizing anti-IL-35 mAb treatment, mouse anti-IL-35 mAb was administered i.p. (70 μg per day, dissolved in 300 μl of PBS) to the NP and AP mice from Day 3 to Day 12 of pregnancy.

After pregnant mice were euthanized, the resorbed (death) embryos were identified by size and necrotic hemorrhagic appearance in comparison with normal embryos. Decidua was isolated and teased apart with the plunger. The collected cells were filtered through 100-μm pores nylon cell strainers. Lymphotypes were isolated by density gradient centrifugation and T_conv_ cells (CD4^+^) were purified using magnetic microbeads (Miltenyi Biotec). Then the cells were cultured in complete medium for further analysis. Animal study was approved by the Animal Investigation Committee of The Second Hospital of Shandong University and we complied with all relevant ethical regulations for animal testing and research.

### Statistical analysis

Statistical analyses were conducted using GraphPad Prism 5 software. Data was presented as mean ± standard deviation (SD). The differences between two groups were assessed using Student’s *t*-test. Serum IL-35 levels in pregnancy and non-pregnant controls were compared using Mann–Whitney *U*-test. A *p* value < 0.05 was considered significant.

### Reporting summary

Further information on research design is available in the Nature Research Reporting Summary linked to this article.

## Supplementary information

Supplementary Information

Reporting Summary

Source Data

## Data Availability

All data generated or analyzed during this study are included in this published article (and its supplementary information files). Source data of the graphs are present in the Source Data file.
