# Supplementary material for: Human placental trophoblast cells contribute to maternal–fetal tolerance through expressing IL-35 and mediating iTR35 conversion
Source: Nat Commun. 2019 Oct 10;10:4601. doi: 10.1038/s41467-019-12484-z (PMC6787064; doi:10.1038/s41467-019-12484-z)
Supplement: Supplementary file 1 — Supplementary Information [file 41467_2019_12484_MOESM1_ESM.pdf]

Supplementary information

Human placental trophoblast cells contribute to maternal-fetal tolerance through expressing IL-35 and mediating iTR35 conversion

Liu et al.

Supplementary Figure 1: Gating strategies used for flow cytometry.

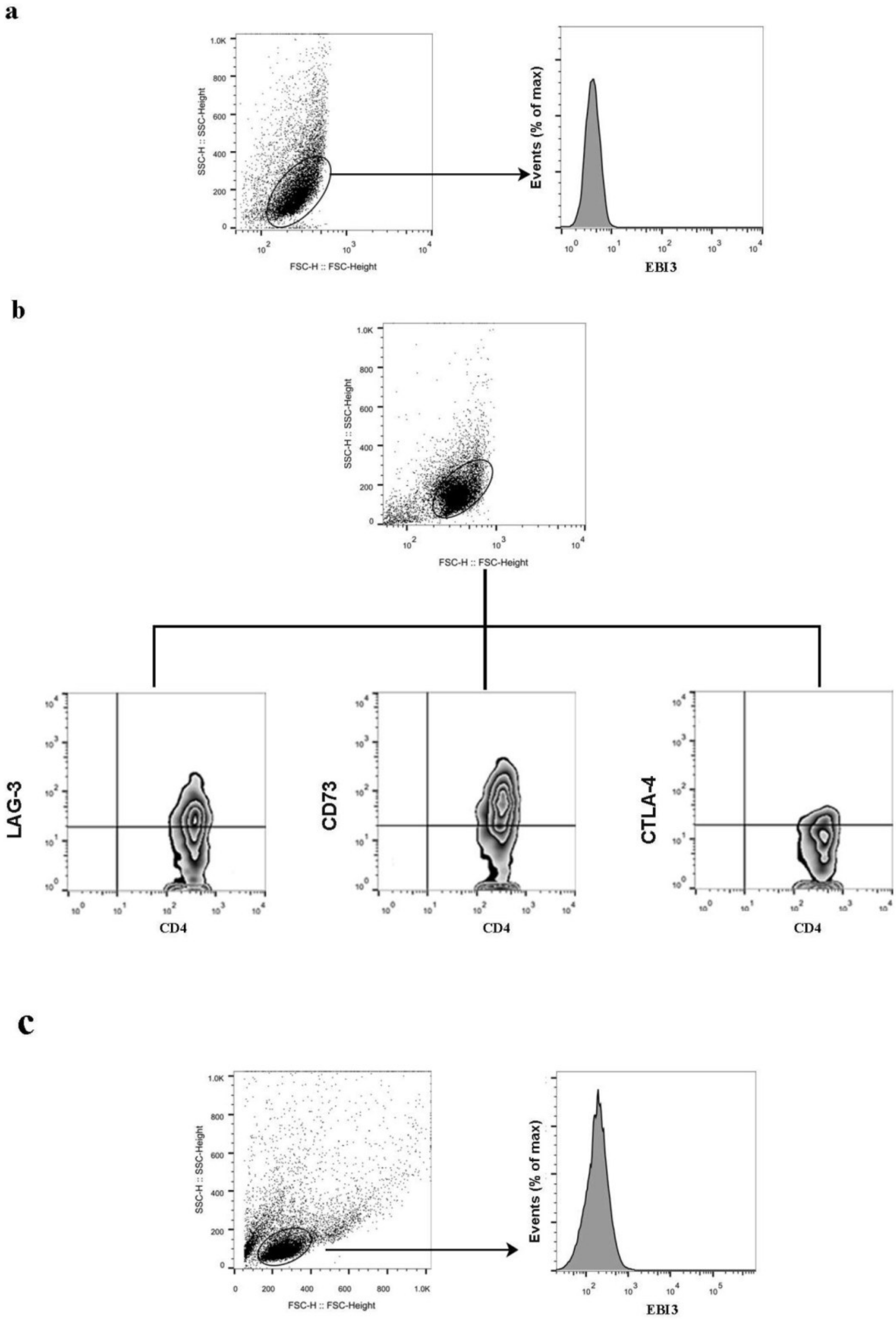

Supplementary Figure 1: Gating strategies used for flow cytometry. a. Gating strategy for detection of EBI3 expression in human T_{conv} cells (figure 2d). b. Gating strategy for figure 3. c. Gating strategy for detection of EBI3 expression in mice T_{conv} cells (figure 6a,6b,6c and 6d).
